# Ecological intensification to mitigate impacts of conventional intensive land use on pollinators and pollination

**DOI:** 10.1111/ele.12762

**Published:** 2017-03-27

**Authors:** Anikó Kovács‐Hostyánszki, Anahí Espíndola, Adam J. Vanbergen, Josef Settele, Claire Kremen, Lynn V. Dicks

**Affiliations:** ^1^ MTA Centre for Ecological Research Institute of Ecology and Botany Lendület Ecosystem Services Research Group Alkotmány u. 2‐4. 2163 Vácrátót Hungary; ^2^ MTA Centre for Ecological Research GINOP Sustainable Ecosystems Group Klebelsberg Kuno u. 3. 8237 Tihany Hungary; ^3^ Department of Biological Sciences Life Sciences South 252 University of Idaho Moscow ID 83844‐3051 USA; ^4^ NERC Centre for Ecology & Hydrology Bush Estate, Penicuik Edinburgh EH26 0QB UK; ^5^ UFZ ‐ Helmholtz Centre for Environmental Research, Dept. of Community Ecology Theodor‐Lieser‐Str. 4, 06120 Halle Germany; ^6^ iDiv German Centre for Integrative Biodiversity Research Halle‐Jena‐Leipzig Deutscher Platz 5e, 04103 Leipzig Germany; ^7^ Institute of Biological Sciences College of Arts and Sciences University of the Philippines Los Banos, College Laguna 4031 Philippines; ^8^ University of California 217 Wellman Hall Berkeley California 94720‐3114 CA USA; ^9^ School of Biological Sciences University of East Anglia Norwich, NR4 7TJ UK

**Keywords:** Crop production, diversification, food security, grazing/mowing intensity, habitat loss, landscape fragmentation, mass‐flowering crops, wild pollinator diversity

## Abstract

Worldwide, human appropriation of ecosystems is disrupting plant–pollinator communities and pollination function through habitat conversion and landscape homogenisation. Conversion to agriculture is destroying and degrading semi‐natural ecosystems while conventional land‐use intensification (e.g. industrial management of large‐scale monocultures with high chemical inputs) homogenises landscape structure and quality. Together, these anthropogenic processes reduce the connectivity of populations and erode floral and nesting resources to undermine pollinator abundance and diversity, and ultimately pollination services. Ecological intensification of agriculture represents a strategic alternative to ameliorate these drivers of pollinator decline while supporting sustainable food production, by promoting biodiversity beneficial to agricultural production through management practices such as intercropping, crop rotations, farm‐level diversification and reduced agrochemical use. We critically evaluate its potential to address and reverse the land use and management trends currently degrading pollinator communities and potentially causing widespread pollination deficits. We find that many of the practices that constitute ecological intensification can contribute to mitigating the drivers of pollinator decline. Our findings support ecological intensification as a solution to pollinator declines, and we discuss ways to promote it in agricultural policy and practice.

## Introduction

The interrelated growth in the global human population, economic wealth, globalised trade and technological developments produces environmental pressures that alter pollinator biodiversity and pollination. The multiple threats to pollinators and plant pollination have been reviewed in detail elsewhere (González‐Varo *et al*. 2013; Vanbergen *et al*. 2013; Potts *et* *al*. 2016a). Recently, the Intergovernmental Science‐Policy Platform for Biodiversity and Ecosystem Services (IPBES) published its ‘Assessment Report on Pollinators, Pollination and Food Production’ (IPBES 2016). This IPBES report globally assessed the status of and threats to pollinators and pollination. It also provided a strategic overview of opportunities to mitigate these threats, thereby safeguarding pollinators and pollination for the future. The IPBES pollinators report clearly identified agriculture as both a threat to pollinators and a potential solution to support them. It also recognised three key complementary policy options for safeguarding pollinators in agricultural ecosystems: adopting ecological intensification, strengthening existing diversified farming systems and building ecological infrastructure. The IPBES report suggested that ‘ecological intensification’ (Bommarco *et* *al*. 2013; Tittonell 2014) is a key mitigation strategy, and claimed it could transform agriculture to support pollinators, pollination services and food production. Its emphasis on managing beneficial biodiversity to maintain or enhance agricultural productivity is particularly appropriate in the context of global policy objectives to achieve greater food security in a changing world (Dicks *et* *al*. 2016a; IPBES 2016). Here, we examine the IPBES report's claim in detail, critically evaluating the potential of ecological intensification to mitigate the negative effects of conventional agricultural intensification (industrial management of large‐scale monocultures with high chemical inputs) on pollinators and pollination, at landscape (land use) and local (land management) scales.

Human land use is the main current driver of changes in land cover (Foley *et* *al*. 2005) on approximately 53% of the Earth's terrestrial surface (Klein Goldewijk *et* *al.,* 2004; Hooke & Martín‐Duque 2012). Globally, since the early 1960s, croplands have expanded with a consequent reduction in forests and grasslands (Klein Goldewijk *et* *al.,* 2004; FAO 2016). By 2030, the area of agricultural land is expected to increase a further 10%, mainly in the developing world (Haines‐Young 2009). These human‐induced changes in land use shift the composition (e.g. habitat loss) and spatial configuration (e.g. fragmentation, isolation) of land‐cover types (Fahrig *et* *al*. 2011). Declines in wild bees and butterflies are linked to historical landscape modification (Burkle *et* *al*. 2013; Bommarco *et* *al*. 2014; Senapathi *et* *al*. 2015) and loss of nesting and foraging sites or key floral resources (Goulson *et* *al*. 2005; Biesmeijer *et* *al*. 2006; Potts *et* *al*. 2010; Scheper *et* *al*. 2014; Baude *et* *al*. 2016). Increasing habitat loss and degradation has also lowered fruit set of insect‐pollinated crops (Klein *et* *al*. 2002, 2012) and wild plants (Aguilar *et* *al*. 2006; Batáry *et* *al*. 2013; Clough *et* *al*. 2014) by eroding pollinator density or diversity. Land‐use changes can also fragment habitats, affecting both the size and connectivity of remnant habitat patches (Hadley & Betts 2012; Hooke & Martín‐Duque 2012). This can potentially reduce pollinator gene flow, with implications for long‐term population persistence (Darvill *et* *al*. 2010), and can adversely affect sexual reproduction of wild plants, particularly of species with an obligate dependence on pollinators (Aguilar *et* *al*. 2006).

Farm management directly affects the availability and quality of foraging and nesting resources for pollinators within agricultural fields (Requier *et* *al*. 2015). Since the 1960s, modern agriculture has rapidly intensified, and the dominant agriculture in many parts of the world now uses large amounts of chemical fertilisers, pesticides, irrigation and other technologies (Tilman *et* *al*. 2001, 2002). Compared with traditional, low‐input farming systems, conventional ‘monocultures’, dominated by one or a few crops, simplify the agroecosystem and decrease pollinator foraging resources (Kremen & Miles 2012).

Despite technological and agronomic improvements, the benefits of conventional agricultural intensification are limited by the available pollination services, at least in pollinator‐dependent crops for which pollination deficits are widely observed (Deguines *et* *al*. 2014; Garibaldi *et* *al*. 2015, 2016b). In recent decades, farming systems and techniques have been developed to mitigate the negative impacts of intensified agriculture on agricultural ecosystems, for example by sustainable intensification, organic farming and agri‐environment schemes (Morandin & Winston 2005; Andersson *et* *al*. 2012; Batáry *et* *al*. 2015). Sustainable intensification originally attempted to increase crop yield while improving ecological and social conditions by the establishment of low‐input ‘resource‐conserving systems’, but recently shifted towards capital and external input intensive solutions to enhance resource‐use efficiencies (Loos *et* *al*. 2014; Garibaldi *et* *al*. 2016a). Organic farming originated to enhance soil fertility, water storage and the biological control of crop pests and diseases. However, recently, certified organic farming also started allowing the controlled use of certain organic pesticides (Garibaldi *et* *al*. 2016a). Thus, today many organic farms are high‐input large‐scale operations supporting low biological diversity (Garibaldi *et* *al*. 2016a). Nevertheless, although the effects of organic farming and other agri‐environment schemes (see also below) on biodiversity – including pollinators – have been mixed, they have been generally positive (Scheper *et* *al*. 2013; Batáry *et* *al*. 2015). Compared to these techniques, ecological intensification describes a process fitting the original concept of sustainable intensification, and aims to support agricultural production through the enhancement of ecosystem services. Its goal is to reduce reliance on anthropogenic chemical inputs in farms, a characteristic it shares with diversified farming systems (cross‐scale diversification, integrating several crops and/or animals in the production system to generate and regenerate ecosystem services) (Kremen & Miles 2012; Bommarco *et* *al*. 2013; Garibaldi *et* *al*. 2016a).

In this paper, we outline the concept of ecological intensification and the ecosystem and agricultural benefits arising from its application. Then – drawing on the findings of the recent IPBES assessment (IPBES 2016) – we consider how land use and land management drivers affect pollinators and pollination. For each set of drivers, we consider whether practices that constitute ecological intensification would reduce or reverse their effects on pollinators and pollination, if widely implemented by farmers. Finally, we discuss the viability and policy implications of ecological intensification as an integrated solution sustaining pollinator communities, pollinator‐dependent crop production and wild plant reproduction.

## What is ‘Ecological Intensification’?

Ecological intensification, as defined by Bommarco *et* *al*. (2013) and Tittonell (2014), involves actively managing farmland to increase the intensity of the ecological processes that support production, such as biotic pest regulation, nutrient cycling and pollination. It means making smart use of nature's functions and services, at field and landscape scales, to enhance agricultural productivity, and reduce reliance on agrochemicals and the need for further land‐use conversion.

We identify specific actions that farmers or land managers may take to achieve ecological intensification (Table 1), including actions focused on enhancing pollination or pest regulation services delivered by mobile agents. Table 1 also summarises the ecosystem services such actions are expected to enhance (adapted from Kremen & Miles 2012), and the potential for mitigating various drivers of change in pollinators and pollination.

**Table 1:** Key practices in Ecological Intensification**,** the ecosystem services they are expected to support (adapted from Kremen & Miles 2012) and how they relate to the main land use and land management drivers of pollinator decline identified by the IPBES pollinators report (IPBES 2016)

Practice	Ecosystem services	Landscape complexity	Landscape connectivity	Nesting resources	Foraging resources	Insecticides and herbicides
Using compost or manure	Soil quality Nutrient management Water‐holding capacity Disease control Energy‐use efficiency Resilience Yield					
Intercropping	Soil quality Weed control Disease control Pest control Pollination Energy‐use efficiency Resilience Yield	+	(+)		+	+
Agroforestry	Soil quality Water‐holding capacity Weed control Disease control Pest control Pollination Energy‐use efficiency Resilience Yield	+	(+)	+	+	
Targeted flower strips	Pest control Pollination Energy‐use efficiency Yield	(+)	(+)		+	(−)
Reduced or no‐till	Soil quality Water‐holding capacity Resilience Yield			+	(+)	
Crop rotation	Soil quality Nutrient management Weed control Disease control Pest control Pollination Energy‐use efficiency Resilience Yield	+			(+)	(+)
Cover crop or green manure	Soil quality Nutrient management Water‐holding capacity Weed control Disease control Pest control Pollination Energy‐use efficiency Resilience Yield			+	+	
Fallow	Soil quality Water‐holding capacity Pest control Pollination Resilience Yield	+	+	+	+	
Border planting (for example, hedgerows and wind breaks)	Nutrient management Pest control Pollination Energy‐use efficiency Resilience	+	+	+	+	+
Riparian buffers	Soil quality Nutrient management Pest control Pollination Energy‐use efficiency Resilience	+	+	+	(+)	+
Patches of semi‐natural zones (woodland, wetland)	Soil quality Nutrient management Pest control Pollination Resilience	+	+	+	+	+
Select crop varieties to enhance recruitment of pollinators or natural enemies	Pollination Pest control				+	(−)
Strategies to reduce pesticide use or exposure of non‐target organisms to pesticides	Pollination Pest control					+
Provide dedicated nesting or overwintering resources for pollinators or natural enemies	Pollination Pest control		(+)	+		

Based on the review here ‘+’ = the driver is reduced or mitigated by this action; ‘(+)’ = the driver may be reduced by this action in some circumstances; ‘−’ = the driver is enhanced or added to by this action; ‘(−)’ = the driver may be enhanced or added to by this action in some circumstances. Blank cells imply the driver is not affected by the action.

Some of the ecological intensification actions identified in Table 1 match existing agri‐environment scheme options or general agricultural conservation measures, such as creating flower‐rich field margins, or managing hedgerows and verges to improve habitat quality. However, the key difference from these other, more biodiversity‐focused approaches is that under ecological intensification these actions would be designed and located to support targeted delivery of ecosystem services. For example, placing flower strips can enhance direct spill‐over of pollination and/or pest regulation services to specific crops in the locality, with their constituent flowering plant species designed to attract and reward specific types of insect (Blaauw & Isaacs 2014a; Tschumi *et* *al*. 2015; Westphal *et* *al*. 2015).

## Land Use, Land Management and Ecological Intensification

The IPBES pollination report (IPBES 2016) assessed the risks and opportunities for pollinators and pollination from land use and land management, amongst other drivers. An expert author and reviewer team of over 70 scientists (including all authors of this paper) critically evaluated the scientific literature, together with evidence provided by indigenous and local knowledge holders, representing all regions of the world. This assessment was peer‐reviewed in an open, two‐stage process by both scientists and governments. It therefore represents a thorough examination of existing global knowledge about pollinators and pollination up to 2016. In this paper, we build on the IPBES report to synthesise the risks to pollinators and pollination at the scales of land use (leading to reduced landscape complexity and connectivity) and land management (leading to reduced nesting and foraging resources within the field). For the latter, we consider arable and grassland systems separately, as the drivers of pollinator decline differ between these habitat types. We also consider agricultural pesticides (insecticides and herbicides) separately because of their high scientific and policy profile as a driver of change in pollinators. For each driver, we assess whether ecological intensification as defined above can ameliorate adverse effects on pollinators and pollination.

## Landscape Complexity and Connectivity

Habitat loss and degradation of habitat quality can reduce the population sizes, composition and species richness of pollinator communities (Steffan‐Dewenter & Westphal 2008; Kennedy *et* *al*. 2013; Ferreira *et* *al*. 2015; Nemésio *et* *al*. 2016) and alter the structure of plant–pollinator networks (Burkle *et* *al*. 2013; Moreira *et* *al*. 2015), with implications for community stability and pollination processes. Specialised pollinator species adapted to particular plant species, or requiring very specific nesting resources (e.g. long‐tongued bumble bees, *Bombus* spp.) tend to be more vulnerable to land‐cover changes than more generalised species (Goulson *et* *al*. 2008; Öckinger *et* *al*. 2010; Weiner *et* *al*. 2014; Persson *et* *al*. 2015). Similarly, above‐ground nesters seem more sensitive to habitat loss or fragmentation than below‐ground nesters (Williams *et* *al*. 2010; Ferreira *et* *al*. 2015; Persson *et* *al*. 2015). Due to different dispersal abilities, different pollinator groups can also show various responses to configurational changes (Redhead *et* *al*. 2016). For instance, small‐bodied pollinators and solitary bees are, owing to their lower dispersal ranges, more vulnerable to effects of habitat fragmentation than larger bodied and social pollinators (Ricketts *et* *al*. 2008; Bommarco *et* *al*. 2010; Williams *et* *al*. 2010; Winfree *et* *al*. 2011). Taken together, these observations identify mechanisms (e.g. filtering by species’ morphological, physiological or ecological traits) explaining the observed homogenisation of pollinator communities in highly human‐modified landscapes (Biesmeijer *et* *al*. 2006; Carvalheiro *et* *al*. 2013; Marini *et* *al*. 2014).

Habitat fragmentation could alter pollinator networks through its effects on pollinator diversity and abundance. Theoretically, by reducing pollinator diversity, fragmentation could reduce pollination network modularity and increase connectance, with small fragments harbouring homogenised pollinator communities (reviewed in Hagen *et* *al*. 2012). The few empirical studies to date suggest fragmentation can reduce nestedness or phylogenetic structure of pollinator networks (Moreira *et* *al*. 2015; Aizen *et* *al*. 2016). Such changes to network structure may have implications for community stability, e.g. by reducing the network's resilience to disturbance (Lever *et* *al*. 2014; Vanbergen *et* *al*. 2017).

For crops, pollination service delivery depends largely on flower visitor density and typically on particular locally abundant pollinator species, which are demographically the least vulnerable to habitat loss or degradation (Kleijn *et* *al*. 2015; Winfree *et* *al*. 2015; Garibaldi *et* *al*. 2016b). Nonetheless, diverse pollinator assemblages offer functional redundancy or complementarity of species or traits to assure resilience of the pollination service (Hoehn *et* *al*. 2008; Klein *et* *al*. 2009; Winfree & Kremen 2009; Garibaldi *et* *al*. 2011, 2016b; Brittain *et* *al*. 2013). Pollination services are affected by the surrounding land use, because it influences both the density and diversity of floral visitors. Blaauw & Isaacs (2014b) showed that the availability of larger patches of floral resources led to higher visitation and wildflower seed set. The delivery of crop pollination services has repeatedly been shown to decrease with increasing distance from florally rich locations (Ricketts *et* *al*. 2008; Garibaldi *et* *al*. 2011; Kormann *et* *al*. 2016). Moreover, production of pollinator‐dependent crops is likely to depend on the relative locations of nests and crops, and on pollinator ability to cover long distances (Osborne *et* *al*. 2008). For example, Sardiñas *et* *al*. (2016) found that ground‐nesting native bees nested both within fields and edges, and that due to short actual foraging distances, visitation by bees nesting in and around fields led to a patchy distribution of pollination services across the field.

Taking this knowledge into consideration, and within the ecological intensification framework (Table 1), restored or maintained semi‐natural ecosystems, field border plantings and riparian buffer strips, can directly increase the complexity and connectivity of agricultural landscapes, often improving floral and nesting resources for pollinator diversity (Lagerlöf *et* *al*. 1992; Carvell *et* *al*. 2004; Cole *et* *al*. 2015; Kremen & M'Gonigle 2015; Baude *et* *al*. 2016; Ponisio *et* *al*. 2016). Optimising management of these semi‐natural areas, such as by adjusting mowing frequency and rotation, can diversify flower and insect pollinator communities (Noordijk *et* *al*. 2009; Halbritter *et* *al*. 2015) across the landscape.

Increasing landscape complexity by employing crop and crop‐livestock mixtures, intercropping and cover crops can increase floral resources and habitat for many pollinator species, even in landscapes with little semi‐natural land‐cover types (Williams & Kremen 2007; Batáry *et* *al*. 2011; Kennedy *et* *al*. 2013). Increasing connectivity by reducing distances between foraging resources elevates pollinator diversity and abundance in fields (Ricketts *et* *al*. 2008; Kennedy *et* *al*. 2013; Clough *et* *al*. 2014). Diversified agricultural systems typified by a large number of crop types and small field sizes (Fahrig *et* *al*. 2015) promote wild pollinator diversity, community stability and pollination success of crops and wild plants (Kremen & Miles 2012; Kennedy *et* *al*. 2013). Polyculture systems with sequentially flowering or co‐flowering crops assure efficient pollination of plants differing in flower phenology by providing seasonal and spatial continuity of food resources supporting pollinator diversity and abundance (Mayfield & Belavadi 2008; Rundlöf *et* *al*. 2014; Ponisio *et* *al*. 2015). Landscape‐scale planning of early and late mass‐flowering crop cultivation, with consideration of the spatial distribution of semi‐natural areas, might aid conservation of pollinators and crop pollination services (Riedinger *et* *al*. 2014). Landscape complexity can also be achieved by temporarily removing land from production (fallow land, Table 1), which can promote the establishment of flower‐rich habitats and the corresponding species richness and abundance of flower‐visiting insects (Morandin *et* *al*. 2007; Kovács‐Hostyánszki *et* *al*. 2011a; Kuussaari *et* *al*. 2011).

Agroforestry, in which large woody perennials are integrated into farming systems, can also increase landscape complexity (Schroth 2004; Willemen *et* *al*. 2013). Agroforestry in temperate systems has been suggested to be favourable to beekeeping (Hill & Webster 1995), and has been estimated to contribute to crop yield and profit through its effect on pollination services (Alam *et* *al*. 2014). In tropical systems, agroforestry is thought to enhance the connectivity of pollinator‐friendly habitats, spatially linking natural and semi‐natural areas (Perfecto & Vandermeer 2008). Further, because in these tropical areas most pollinators rely on tree flowers (Bawa 1990), agroforestry practices could contribute to pollinator conservation, as seen in coffee and cacao plantations. Through its effect on pollinator abundance, proximity to forested areas increases yield (Klein *et* *al*. 2002; Ricketts 2004), as well as resilience and stability of pollinator communities (Bravo‐Monroy *et* *al*. 2015). Further, tree diversity and cover positively correlate with native bee abundance (Klein *et* *al*. 2002; Jha & Vandermeer 2010) and richness (Hoehn *et* *al*. 2010), and a link was found between low‐impact management, in‐field bee diversity and crop pollination (Vergara & Badano 2009).

## Land Management to Increase Local Nesting and Foraging Resources

### Crop fields

Many traditional systems of land management encompass cultivation of sequentially‐ and co‐flowering crops alongside high wild plant diversity, with low agricultural inputs and low yields (Plieninger *et* *al*. 2006). These systems usually favour pollinator biodiversity (Stoate *et* *al*. 2001; Kovács‐Hostyánszki *et* *al*. 2016). Although still largely present in many parts of the world (Altieri *et* *al*. 2012), traditional systems have today mostly disappeared in Europe and North America due to abandonment or conventional intensification of land management (Stoate *et* *al*. 2001; Fontana *et* *al*. 2014; Norfolk *et* *al*. 2014; Kovács‐Hostyánszki *et* *al*. 2016).

In contrast to diversified farming systems, monocultures reduce landscape complexity (see above) and overall habitat resources for pollinators, despite the provision of alternative foraging resources by certain mass‐flowering crops. Monocultures of crops such as canola (*Brassica napus*), clovers (*Trifolium* spp.), sunflowers and orchard fruits provide large amounts of accessible pollen and nectar, which has been shown to benefit bee colonies (e.g. increased densities, reproductive success) (Klein *et* *al*. 2007; Westphal *et* *al*. 2009; Diekötter *et* *al*. 2010, 2014; Holzschuh *et* *al*. 2013; Riedinger *et* *al*. 2015). However, with few other floral resources in such intensively managed fields, the temporary synchronous pulse of pollen and nectar from such crops means that benefits are transient and limited to the duration of crop flowering (Blitzer *et* *al*. 2012; Riedinger *et* *al*. 2015). Furthermore, any benefits of mass‐flowering crops mostly affect generalist pollinators and their pollination services at the cost of wider pollinator diversity (Jansson & Polasky 2010; Holzschuh *et* *al*. 2011). However, the cultivation of plants flowering late in the season (e.g. red clovers) can extend the temporal supply of foraging resources after the bloom of other crops to enhance bumble bee reproductive success (Rundlöf *et* *al*. 2014). The massive bloom of mass‐flowering crops can also temporarily dilute landscape pollination services by diverting pollinators from co‐flowering wild plants (Stanley & Stout 2014; Holzschuh *et* *al*. 2016; Montero‐Castaño *et* *al*. 2016) to reduce pollination (Holzschuh *et* *al*. 2011; Montero‐Castaño *et* *al*. 2016), and potentially negatively affect crop yield and wild plant reproduction (Kovács‐Hostyánszki *et* *al*. 2013; Holzschuh *et* *al*. 2016).

Besides flowering crops, weed flowers provide a diversity of foraging resources for wild and managed pollinators (Carvalheiro *et* *al*. 2011; Bretagnolle & Gaba 2015; Requier *et* *al*. 2015). Thus, their removal by physical means (e.g. tillage, crop rotation) or by herbicides (see ‘Insecticides and herbicides’ section) can indirectly cause the decline of pollinator populations (Richards 2001; Steffan‐Dewenter *et* *al*. 2005; Diekötter *et* *al*. 2010). The increased application of inorganic fertilisers (Richards 2001) can also reduce the diversity and cover of the less competitive wild and weedy plant species (Kleijn *et* *al*. 2009; Kovács‐Hostyánszki *et* *al*. 2011b). Further, nitrogen fertilisation may also change the number, size, morphology, nectar chemistry and phenology of flowers, thereby altering plant–pollinator mutualisms (Burkle & Irwin 2010; Hudewenz *et* *al*. 2012), and can also decrease crop yield due to insect pollination (Marini *et* *al*. 2015).

Existing agri‐environment measures that benefit pollinator richness by providing habitats and food resources within and around fields can be deployed as part of a shift towards ecological intensification (Nicholls & Altieri 2013; Dicks *et al*. 2014a, 2014b). These practices include establishment of flower‐rich areas such as sown field margins, flower strips, hedgerows and fallow fields (Table 1). Although population‐level effects on pollinators are yet to be detected, flower strips rich in nectar or pollen increase the production of bumble bee reproductives (Williams *et* *al*. 2012; Carvell *et* *al*. 2015). Furthermore, they elevate local pollinator abundance and diversity compared to sown grass margins, natural regeneration or cropped areas (Carvell *et* *al*. 2007; Scheper *et* *al*. 2013). Hedgerow plantings can also increase wild bee richness and persistence within crop fields (M'Gonigle *et* *al*. 2015) as well as turnover (regional richness) among fields (Ponisio *et* *al*. 2015). However, the pollination service benefits may be crop‐ and region‐specific (Sardiñas & Kremen 2015; Morandin *et* *al*. 2016) and vary with farmland type and landscape context (Scheper *et* *al*. 2013, 2015).

Designing flower mixes to provide continuous bloom throughout the growing season, and including functionally diverse species (i.e. perennials and annuals) is critical to supporting the greatest pollinator species richness within these established flower‐rich areas (Scheper *et* *al*. 2015; Williams *et* *al*. 2015). Regional programs such as Operation Pollinator run by Syngenta, have developed and tested seed mixtures for land managers (Williams *et* *al*. 2015; http://www.operationpollinator.com/). However, flower‐rich plantings often provide resources mainly for bumble bees and honey bees, while the majority of other pollinator species cannot find their favoured flower species (Wood *et* *al*. 2016). Wildflower strips can increase pollinator abundances across entire landscapes, especially in landscapes dominated by intensive farmland (Jönsson *et* *al*. 2015). Similarly, hedgerows can also increase local (M'Gonigle *et* *al*. 2015) and regional pollinator species richness (Ponisio *et* *al*. 2015).

Although their impact on pollinator diversity and abundance is accepted, there is still limited evidence about the direct impact of wildflower strips, flower patches and hedgerows on crop yields. In the case of wildflower strips, some studies demonstrate increased pollination of adjacent crops [e.g. mango in South‐Africa (Carvalheiro *et* *al*. 2012); blueberry in USA (Blaauw & Isaacs 2014a); strawberry in UK (Feltham *et* *al*. 2015)], while another showed that flower strips increase outcrossing with adjacent crops, affecting the genetic structure of the cultivar (Suso *et* *al*. 2008). Regarding hedgerows, Morandin *et* *al*. (2016) found a benefit to pollination in the adjacent crop, whereas Sardiñas & Kremen (2015) found no difference. Planting wildflower strips potentially increases the risk of favouring herbivore pests (Holland *et* *al*. 2016), but this could be minimised by including diverse perennial flowering plants and increasing the size of the plot. This way, more beetles, birds and other predators are also attracted and can aid with biological pest control (Blaauw & Isaacs 2012; Tschumi *et* *al*. 2015; Westphal *et* *al*. 2015; Morandin *et* *al*. 2016; Sidhu & Joshi 2016).

Ecological intensification in arable fields has been demonstrated to enhance within‐field species richness and abundance of plants considered weeds, by decreasing the use of inorganic fertilisers and herbicides (Kleijn *et* *al*. 2009; Kovács‐Hostyánszki *et* *al*. 2011b). If crop and weed species are left to grow together, diverse pollinator assemblages benefit crop pollination both in arable fields (Carvalheiro *et* *al*. 2011) and orchards (Alaux *et* *al*. 2010; Holzschuh *et* *al*. 2012; Saunders *et* *al*. 2013; Cierjacks *et* *al*. 2016; Kammerer *et* *al*. 2016; Norfolk *et* *al*. 2016). Further, weed flowers have been shown to mitigate the negative effects of certain types of land management and/or the isolation from natural land‐cover types (Carvalheiro *et* *al*. 2011, 2012), and sustain pollinator assemblages after or between the mass‐flowering crop periods (Requier *et* *al*. 2015). Within‐field diversification can be even more effective if wild flower patches and a diverse landscape structure are available nearby or around the managed sites (Kennedy *et* *al*. 2013).

Wild pollinator communities within crop fields can also be enhanced by providing nesting material or available nesting sites, which are often scarce in conventional intensive management systems. Conditions for cavity‐nesting and ground‐nesting bees can be improved by providing natural or artificial nest substrates such as reed stems, muddy spots and bare ground at crop edges (Sheffield *et* *al*. 2008; Steffan‐Dewenter & Schiele 2008). Combining this with abundant floral resources was found to increase pollinator population growth (Oliveira Filho & Freitas 2003), but there is currently little evidence that such nesting site practices increase yield of adjacent crops (Camillo 1996).

As part of conservation agriculture practices, no‐till farming is applied for soil conservation, and can reverse long‐term soil degradation due to organic matter loss, although a major constraint on its adoption is the limited availability of agricultural training (Chan & Fantle‐Lepczyk 2015). A drawback of no‐till practice is a potential reduction in yields, although this risk can be minimised by combining it with crop rotation and measures to retain crop residue *in situ*. No‐till significantly increases rain‐fed crop productivity in dry climates (Pittelkow *et* *al*. 2015). There is still no strong evidence for a positive effect of no‐till farming on ground‐nesting pollinator species (Shuler *et* *al*. 2005), although this has often been proposed, since many species place their brood cells < 30 cm below the surface (Williams *et* *al*. 2010; Cane & Neff 2011; Roulston & Goodell 2011; Ullmann *et* *al*. 2016). Besides through its direct effect on the soil, sustained tillage coupled with herbicide use reduce the availability of weeds that provide in‐field floral resources for pollinators. A possible disadvantage of no‐till farming for pollinators may arise if farmers are compelled to increase herbicide use to counter burgeoning weed populations. This potential outcome and the relative reductions in floral resources for pollinators compared with conventional and low‐input or organic farming systems remain to be determined (McLaughlin & Mineau 1995).

Using compost or manure to improve soil organic matter content and long‐term soil fertility as part of a nutrient management plan is the only ecological intensification action for which there is not a clear link to drivers of pollinator decline. It could conceivably affect nesting resources for ground‐nesting wild bees, but the direction of this impact is unclear and we are unaware of any studies to date. There are, however, some studies demonstrating that alterations in soil nutrients and microorganisms can affect floral characteristics that may influence flower visitation and pollinator nutrition (Cardoza *et* *al*. 2012; Barber & Gorden 2014).

### Grasslands

Grazing livestock and mowing alter ecosystems, affecting wild plant reproduction and the amount of floral resources available to pollinators (Mayer 2004; Wesche *et* *al*. 2012; Vanbergen *et* *al*. 2014a). Grazing affects pollinators and pollination in complex ways (Ågren *et* *al*. 2013) that depend on the grazing intensity, selectivity, timing, climate, habitat type, etc. (Asner *et* *al*. 2004; Kimoto *et* *al*. 2012; Tadey 2015). For instance, the intensity of herbivory can shape the attractiveness of flowers to pollinators (Ågren *et* *al*. 2013), with highly intensive grazing able to lower forb coverage or diversity with concomitant impacts on pollinator densities, diversity and network structure (Kruess & Tscharntke 2002; Yoshihara *et al*. 2008; Potts *et* *al*. 2009). Grazing livestock (e.g. cattle, sheep) results in soil compaction by trampling, and affects the amount of nesting resources available to pollinators, influencing their abundance or diversity (Mayer 2004). However, moderate grazing can increase pollinator diversity and complexity of pollinator networks by altering plant communities (Vulliamy *et* *al*. 2006; Vanbergen *et* *al*. 2014a; Lázaro *et al*. 2016). Further, spatial planning and grassland management that increases spatial heterogeneity in the grazed area can be beneficial to pollinator diversity in regions adapted to grazing, such as those historically grazed by native large herbivores (Fuhlendorf & Engle 2001).

Although mowing results in sudden removal of almost all foraging resources for pollinators, its frequency and timing also influence the composition of vegetation over time (Forrester *et* *al*. 2005; Nakahama *et* *al*. 2016), thus indirectly affecting pollinator diversity and abundance (Rasmont *et* *al*. 2006). Frequent mowing during a growing season reduces native plant growth and the ability of forbs to compete with grasses (Gerell 1997; Saarinen *et* *al*. 2005), impoverishes plant‐visitation networks (Weiner *et* *al*. 2011), and can negatively affect bumble bee populations by eliminating preferred leguminous pollen resources (Osborne *et* *al*. 1991; Goulson *et* *al*. 2005). Mowing can cause direct pollinator mortality, particularly of egg and larval stages (Di Giulio *et* *al*. 2001; Humbert *et* *al*. 2010), it can remove butterfly host plants (Johst *et* *al*. 2006), and destroy topographical features such as grass tussocks (Morris 2000) that offer potential nesting sites for bumble bees (Hopwood *et* *al*. 2015). It can also disturb ant nests, which in turn affects the survival of myrmecophilous butterflies (Wynhoff *et* *al*. 2011).

Of all ecological intensification actions listed in Table 1, flower strips, riparian buffer strips and border plantings are the easiest‐to‐apply options to transform grassland management. Along with these, many subtle changes in management can enhance ecosystem services and could be included in an ecological intensification framework. For example, optimising the timing and frequency of mowing can benefit communities of flowers, pollinators, and other organisms such as nesting game birds. Specifically, flower‐visiting insects benefit from a biannual cut with hay removal, where an early summer cut enables the re‐flowering of plants later in the growing season (Noordijk *et* *al*. 2009). Mowing without a conditioner, and refraining from mowing in periods of increased flight activity, are two practices strongly recommended to reduce pollinator mortality (Humbert *et* *al*. 2010), while leaving uncut refuges and delaying mowing help to further mitigate the impact on pollinators (Humbert *et* *al*. 2012; Buri *et* *al*. 2013). Regular (but not too frequent) mowing is also necessary to maintain moderate sward height, for example, for ants associated with myrmecophilous butterflies (Settele & Kühn, 2009). Switching to periodic mowing, combined with manual extraction of tall vegetation and use of selective herbicides, was shown to increase the diversity of bee communities and number and distribution of rare pollinator species along powerlines (Russell *et al*. 2005). Improved grasslands can be managed for floral richness, by reducing fertiliser inputs or delaying mowing dates, which concomitantly increases the bee, hoverfly and/or butterfly diversity (Humbert *et* *al*. 2012; van Swaay *et* *al*. 2012; Dicks *et al*. 2014a, 2014b). Adding legumes and other flowering species to grassland seed mixtures supported by some agri‐environmental schemes in Europe can probably further benefit pollinators (Dicks *et* *al*. 2010, 2015; Dicks *et al*. 2014a, 2014b; Woodcock *et* *al*. 2014).

## Insecticides and Herbicides

The use of insecticides represents a hazard to pollinator health, diversity and abundance. However, the risk varies with the toxicity of the particular insecticide to different species and by the level of exposure according to management practice, and the phenology, behaviour and habitat use of foraging pollinators (Brittain & Potts 2011; Schreinemachers & Tipraqsa 2012; Godfray *et* *al*. 2014, 2015; van der Sluijs *et* *al*. 2015; IPBES 2016). Aside from mortality, insecticides can elicit a range of sublethal effects on pollinators, such as physiological and behavioural changes (Godfray *et* *al*. 2014, 2015; Pisa *et* *al*. 2015). Most of our knowledge on insecticide effects comes from laboratory or semi‐field experiments: where insects are dosed in the laboratory and then forage freely in the wild (Godfray *et* *al*. 2014, 2015). There are relatively few controlled field experiments assessing actual exposure to insecticides in field settings (Godfray *et* *al*. 2014, 2015). A notable example is a recent farm‐scale replicated experiment, which found that actual field exposure to oil‐seed rape treated with the neonicotinoid clothianidin and pyrethroid insecticides decreased survival and reproduction of wild bee species, relative to oilseed rape treated only with pyrethroids (Rundlöf *et* *al*. 2015). How lethal and sublethal effects of insecticides affect colonies and populations of managed and wild pollinators over the long term remains unclear (IPBES 2016). However, a recent historical population analysis of the population persistence of pollinators associated or not with neonicotinoid treated oilseed rape crops indicated that the former present slight, but statistically significant, reductions (Woodcock *et* *al*. 2016). The role of insecticides in affecting pollination services is unclear, but there is some new experimental evidence that insecticides affect floral preference and thus can modify the likelihood of delivery of pollination services to crop and wild plants by altering bee behaviour (Stanley *et* *al*. 2015; Stanley & Raine 2016).

Beside insecticides, the increased use of synthetic herbicides (Schwinn 1988; Freemark & Boutin 1995) can threaten pollinator populations by reducing floral resources (Richards 2001; Steffan‐Dewenter *et* *al*. 2005; Diekötter *et* *al*. 2010). This is true not only for in‐field floral resources but also for those present in field margins, which can be exposed to herbicide drift from adjacent crops (Egan *et* *al*. 2014; Hanley & Wilkins 2015; Bohnenblust *et* *al*. 2016). Moreover, some herbicides can be directly toxic to larval stages of pollinators (Hahn *et* *al*. 2015). Weed‐removal is at the core of management of herbicide‐tolerant GM‐crops and while comparatively understudied, these crops can be associated with lower pollinator densities (Bohan *et* *al*. 2005; Bohnenblust *et* *al*. 2016). Their widespread and expanding cultivation could represent a threat to pollinators, increase pollination deficits and yield reduction in insect‐dependent crops, but this needs further study.

Several measures within the scope of ecological intensification (Table 1) can reduce the risk to pollinators from pesticides. Reductions in overall use or reliance on pesticides reduce the risk to non‐target species like pollinators (IPBES 2016). Planting buffer zones or wind breaks at field borders and agricultural technologies (e.g. low‐drift spraying equipment) can reduce pesticide drift into adjacent habitats (Ucar & Hall 2001; Felsot *et* *al*. 2010). Adopting alternative forms of pest control can also help lower insecticide risks. For instance, establishing or maintaining semi‐natural areas or hedgerows around the managed fields (Table 1) can support biological control agents that may enhance pest predation in the adjacent crops, reducing the need for pesticide use (Denys & Tscharntke 2002; Morandin *et* *al*. 2014). Furthermore, such ecological infrastructure can provide alternative nesting and foraging habitats for pollinators in the landscape, thereby buffering insecticide and herbicide effects in the crop field (Park *et* *al*. 2015). However, these field margins, particularly if planted with forage resources, could act as ecological traps for pollinators potentially increasing their exposure to insecticides used in the adjacent crop (Longley & Sotherton 1997).

In conventional and organic farming, adoption of integrated pest management (IPM) is suggested to be the best effort to decrease pesticide use, and is very likely to form part of ecological intensification strategies. IPM decreases the need for pesticides through a greater reliance on biological pest control and managing pest pressure through crop rotation, mixed cropping and field margin or habitat management (Table 1). Pesticides are applied in a targeted way only when other measures are insufficient to hold pest abundances below a threshold of economic damage (Ekström & Ekbom 2011; USDA 2014). In order to reduce insecticide use and promote non‐chemical pest management practices, the EU obliged all Member States to apply the general principles of IPM by 2014 (Directive 2009/128/EC). Farmers can also reduce exposure of pollinators to pesticides using farm‐scale risk assessment and mitigation. For example, a tool that assesses risk from pesticide exposures to pollinators in the field has already been developed (Van der Valk *et* *al*. 2012), using local information about crop pollinators, while listing the main factors affecting pesticide risk (e.g. pesticide type and use, phenology of crop flowering and pollinator activity).

To summarise, we suggest that most facets of ecological intensification, whether focused on pollination or other ecosystem services, can address one or more of the major land use‐related drivers of pollinator decline (Table 1). However, there remain serious questions about whether ecological intensification is a viable approach for commercial farming, and about how ecological intensification should be incentivised within current agricultural and agri‐environmental policies. Below, we briefly discuss these issues.

## Viability of Ecological Intensification in Farming

The viability of ecological intensification may be challenged either on the basis that its outcomes are uncertain and unpredictable, or that it might not be able to achieve levels of crop production equivalent to conventional agricultural intensification (Garibaldi *et* *al*. 2016b).

### Level of uncertainty

Broadly, when using ecological intensification to improve the sustainability of farming, uncertainties stem from two sources: scientific or agronomic uncertainty on how to implement ecological intensification, and the inherent unpredictability of natural systems (stochastic uncertainty). The efficacy of ecological intensification as a process to maintain or enhance current levels of food production relies on efficient and consistent ecosystem service delivery. However, there is not yet scientific consensus about the structure of service provider communities required to achieve this. On this, Kleijn *et* *al*. (2015) suggest that a limited number of generalist wild bee species are responsible for the majority of pollination of the 100 most important insect‐pollinated crop species globally. However, other analyses show that resilience of pollinator communities over time, and the stability and resilience of pollination services and crop productivity strongly rely on pollinator diversity, complementarity and redundancy (Blüthgen & Klein 2011; Brittain *et* *al*. 2013).

### Level of overall productivity

The potential for ecological intensification to maintain production was highlighted in a farm‐scale multi‐year field experiment where up to 8% of land was removed from production to create wildlife habitat (e.g. sown patches of perennial native wildflowers and fine‐leaved grasses mix) (Pywell *et* *al*. 2015). The results suggested that this management shift over a 6‐year period can lead to no net loss of overall monetary value or nutritional energy of crops produced at the whole‐farm level, as a result of increases in per‐unit area productivity. Specifically, the yield of the insect‐pollinated crop field bean *Vicia faba* increased by 25% and 35% in the case of 3% and 8% wildlife habitat establishment, respectively (Pywell *et* *al*. 2015). In this study, it took around 4 years for the beneficial effects on crop yield to manifest, probably reflecting the time taken for populations of pollinators and other beneficial insects to respond to ecological intensification. This result also illustrates another challenge for implementing ecological intensification – there is likely to be a transition period, when overall productivity may be lower. However, a recent review found that transitioning yield losses can be negated or eased by using more crop diversification practices (Ponisio & Ehrlich 2016).

## How to Promote or Support Ecological Intensification in Policy

Most current approaches to support environmentally friendly farming assume an income loss for the farmer, who should be compensated or motivated by financial support to implement such practices. For example, costs associated with the establishment and maintenance of practices such as flower‐mix plantings can be a major hurdle in their successful adoption (Sidhu & Joshi 2016). In Europe, the USA and Australia, agri‐environment schemes (AES) offer farmers short‐term payments or cost‐share for performing prescribed environmental management behaviour. Initially, the purpose of AES in the USA was to conserve soil and prevent erosion, while in Europe it aimed at protecting threatened habitats or landscapes. However, emphasis in the USA and Europe later broadened to cover prevention of species’ loss across agricultural landscapes, and, more recently, to maintain ecosystem services (Ekroos *et* *al*. 2014; Dicks *et* *al*. 2016b). Recently, it was suggested that switching to ‘payment by results schemes’ (i.e. paying farmers for outcomes rather than for performing set management activities) could be an effective instrument for changing farming social behaviour, and could encourage the establishment of common goals between farmers and conservationists (de Snoo *et* *al*. 2012; Magda *et* *al*. 2015). However, these schemes can potentially punish farmers who act adequately but do not achieve the goals because of external variables, such as land configuration (e.g. their fields are too isolated from the closest pollinator habitats; Brittain *et* *al*. 2010). Payment for ecosystem services (PES) could promote practices to conserve pollinators on farms too (e.g. Daily *et* *al*. 2009). Other potential paths could also include the labelling and production of ‘pollinator‐friendly’ foods, as well as promoting ecological intensification with food‐producing and retail corporations.

In ecological intensification, while there may be a transition period during which the costs outweigh the benefits (see above), actions are expected to support production and should not entail an overall cost in the longer term. As a result, the framework of compensation for lost income that is embodied in AES does not apply so easily. ‘Compulsory greening measures’ under the direct payments pillar of the EU's Common Agricultural Policy from 2014–2020 represent an alternative approach where payments are not designed to cover lost income, but environmental actions are required as a condition of subsidy payments. These payments incorporate several potential elements of ecological intensification across Europe, such as field margins, buffer strips and fallow land, through an obligation to keep 5% of arable lands as ‘Ecological Focus Areas’ (Pe'er *et al*. 2016; Tzilivakis *et al*. 2016; Table 1). There is a great opportunity now to use the Ecological Focus Areas policy to promote targeted measures effective at enhancing specific ecosystems services such as pollination (Dicks *et al*. 2014a, Pe'er *et* *al*. 2016; Tzilivakis *et al*. 2016; Table 1).

In the USA, conservation measures financed by the Farm Bill, such as the Environmental Quality Incentives Program (EQIP) can also fit with the notion of ecological intensification. EQIP programs provide cost‐share funding (generally 50% of the cost of implementation) that incentivise a wide array of sustainability and conservation measures on farms, including a number that are specifically designed to support pollinators, such as planting forb strips and flowering hedgerows along field margins. For these measures, studies have shown that the farmers’ cost‐share portion would be balanced by the yield benefits received within 4 and 7 years, respectively (Blaauw & Isaacs 2014a; Morandin *et* *al*. 2016). Other Farm Bill programs, such as the Conservation Reserve Program (CRP), pay farmers to retire sensitive lands and restore them for selected functions, including restoring pollinator habitat. The 2016 National Strategy on Pollinator Health sets a goal of conserving, restoring or enhancing land for pollinators, through a variety of Farm Bill conservation measures.

Further research, extension and infrastructure investment are needed to demonstrate how to reach greater yields and profits with ecological intensification (Parmentier 2014; Ponisio *et* *al*. 2015; DeLonge *et* *al*. 2016; Garibaldi *et* *al*. 2016b). Training and education of farmers and agronomists are also essential to increase the effectiveness of ecological intensification, including those elements focused on pollinators and pollination (Lobley *et* *al*. 2013). Policies that support farmer field schools are known to increase the adoption of IPM practices and farmers’ income by reductions in insecticide use (Waddington *et* *al*. 2014). Related to this, the dialogue among farmers, scientists and policy‐makers, and accounting for farmers’ insights in decision making, are valuable to understand and address different perspectives and needs, and confer higher quality decisions and greater legitimacy (Menzel & Teng 2010; Garibaldi *et* *al*. 2016b).

## Knowledge Gaps and Future Directions

In this review, we identified different knowledge gaps, which we grouped under three main topics:

### The effect of land use and landscape drivers on the long‐term survival of pollinator communities

A lot of research has characterised the effects of specific land management and land‐use changes on the abundance or diversity of different pollinator groups (Kennedy *et* *al*. 2013; Bommarco *et* *al*. 2014). However, long‐term impacts of these land changes on pollinator and plant community structure, pollination networks and pollinator demography are little understood. For instance, few studies have empirically investigated how pollination networks are affected by changes in landscape (Moreira *et* *al*. 2015; Aizen *et* *al*. 2016), or evaluated the effect of these changes on the long‐term survival and evolutionary potential of affected pollinator species. Better understanding these ecological processes is key for predicting the responses of pollination communities to land use and landscape changes.

### The effect of changes in the pollinator community on crop yield and the reproductive success of wild plants

Scientists are only starting to understand how changes in pollinator communities affect pollination services (Vanbergen *et* *al*. 2014b). For example, although it has been shown that more pollinators increase yield (Garibaldi *et* *al*. 2011, 2015), it remains unclear whether benefiting generalist pollinators is sufficient to secure crop and wild plant yields. On the same lines, there is no clarity about what level of pollinator diversity or even pollinator abundance is the minimum required to maintain healthy and diverse plant communities, and high crop yield and quality. This knowledge is needed to support efficient application of ecological intensification measures, and a more informed decision‐making process.

### Communicating knowledge and translating it into policies and actions

Although farmer education and knowledge dissemination have proved effective in applying ecological intensification, these tools appear to be underexploited. To improve this, a better integration of local knowledge and culture into educational programs would largely increase the use and spread of ecological intensification (Geertsema *et* *al*. 2016). Further, improving our understanding of the financial costs, benefits, return on investment, and how stable those returns are is critical for a successful application of ecological intensification. The integration of cultural, economic and ecological knowledge was successful and fruitful in the global IPBES pollination assessment (IPBES 2016), but similar approaches should also be used at local and regional scales. Doing this will require further integrating and complementing studies on the sociological, economic and ecological aspects of food production and agriculture.

## Conclusion

In this review, we argue that ecological intensification has the potential to support pollinators by bringing ecosystem services into crop production systems, and replacing chemical inputs (Bommarco *et* *al*. 2013). However, there is still a long way to reach optimal management in ecologically intensified systems and in filling current knowledge gaps. We describe research supporting a positive impact of most elements of ecological intensification on pollinator diversity and abundance. We suggest that landscape‐scale (instead of farm‐scale) management of agricultural areas could result in better provisioning of pollination services by improving habitat availability and configuration. Such landscape management decisions, however, need proper coordination of farmers’ land‐use decisions (Plieninger *et* *al*. 2012; Cong *et* *al*. 2016). Along with this, and to inform these decisions, we have identified some knowledge gaps that need to be addressed.

Regionally or locally tailored solutions are needed for effective ecological intensification. Further, in order to achieve ecological intensification goals, institutional and productivity innovations (e.g. better access to credit and market, better knowledge on agricultural production techniques; Schut *et* *al*. 2016) are needed, particularly in developing countries. Finally, ecologists, commercial agronomists and extension workers need to play the central role of facilitating ecological intensification by engaging with stakeholders such as farmers, government agencies and environmental NGOs. This engagement includes working on filling knowledge gaps by generating knowledge to support decision making, and applying this knowledge to develop sound solutions for farm and landscape management (Geertsema *et* *al*. 2016). To reach such applications, the integration of agricultural, ecological, economic and social sciences will be central to developing the sustainable agriculture needed to fulfil the UN sustainable development goals.

## Authorship

AKH and AE wrote the first draft of the manuscript and all authors contributed substantially to the final manuscript, provided corrections to manuscript drafts, and discussed ideas within it.

## Data Availability

No unpublished data were used in the review.
